# Prolonged Deleterious Influences of Chemotherapeutic Agent CPT-11 on Resident Peritoneal Macrophages and B1 Cells

**DOI:** 10.3389/fimmu.2017.01919

**Published:** 2018-01-05

**Authors:** Wen-Jing Bai, Chen-Guang Li, Cheng-Cheng Zhang, Li-Hui Xu, Qiong-Zhen Zeng, Bo Hu, Zhou Hong, Xian-Hui He, Dong-Yun Ouyang

**Affiliations:** ^1^Department of Immunobiology, College of Life Science and Technology, Jinan University, Guangzhou, China; ^2^Department of Cell Biology, College of Life Science and Technology, Jinan University, Guangzhou, China; ^3^Department of Nephrology, The First Affiliated Hospital of Jinan University, Guangzhou, China

**Keywords:** CPT-11, resident peritoneal macrophages, peritoneal B1 cells, cytotoxicity, adoptive transfer

## Abstract

CPT-11 is a first-line chemotherapeutic agent for the treatment of colorectal cancer in clinic. Previous studies including ours have demonstrated that CPT-11 is, however, toxic to the intestinal epithelium and resident peritoneal macrophages. By interacting with B1 cells, the resident peritoneal macrophages play critical roles in the maintenance of gastrointestinal homeostasis. It remains therefore elusive whether these peritoneal innate immune cells could be rebuilt spontaneously or artificially after being impaired by CPT-11 administration. In this study, we found that mouse resident peritoneal macrophages, namely the large peritoneal macrophages (LPMs) with a CD11b^+^F4/80^hi^GATA6^+^ phenotype, and B1 (CD19^+^CD23^−^) cells were depleted by intraperitoneal (i.p.) CPT-11 treatment within 1 week, but reappeared from day 14 after CPT-11 treatment. However, the recovery processes of these innate immune cells were slow, as their counts could not be fully recovered even 2 months later, when compared with that of vehicle-treated control group. Interestingly, in the peritoneal cavity of the mice treated with CPT-11, the cell counts of LPMs and B1 cells were significantly increased after adoptive transfer with syngeneic peritoneal exudate cells (PECs) from healthy mice. Adoptive transfer with bone marrow cells also slightly increased, although not significantly, the cell counts of LPMs and B1 cells in CPT-11-treated mice. The survival rate of bacterial infected mice was significantly reduced by i.p. CPT-11 treatment in comparison with vehicle-treated or untreated control groups. Besides, oral administration of CPT-11 also had a delayed toxicity on the resident peritoneal macrophages. Our results suggest that CPT-11 has prolonged deleterious effects on peritoneal innate immune cells but adoptive transfer with PECs may accelerate their recovery processes, highlighting the potential of adoptive cell transfer as an avenue to counteract the adverse effects of this chemotherapeutic agent.

## Introduction

There are several types of immune cells in the exudate of the peritoneal cavity, including macrophages and B cells, which play critical roles in defense against infections and in maintaining homeostasis of tissues where they reside (1, 2). Emerging evidence has indicated the phenotypic and functional diversities of these tissue-resident immune cells. For example, recent studies revealed that peritoneal macrophages are heterogeneous (3, 4). In mice, they can be further classified into two subpopulations according to their cell surface expression of F4/80 and MHC class II molecules. One subpopulation is constituted by large peritoneal macrophages (LPMs) with high levels of F4/80 but low levels of MHC class II, while the other is composed of small peritoneal macrophages (SPMs) with low levels of F4/80 but high levels of MHC class II (3). These two subpopulations are not only different in phenotypes but also in functions (3). It is generally believed that the LPMs are tissue-resident macrophages which are developed from yolk sac and are self-renewing, whereas the origin of SPMs is not quite clear (5, 6). Some studies have observed that the MHC II^+^ SPMs can be replenished by circulating monocytes (7); but others have indicated that SPMs are not present in the blood and they are phenotypically distinct from monocytes (6). Apart from macrophages, B cells in the peritoneal exudate are also heterogeneous (8). Both B1 and B2 cells are included, but the ratio of B1 cells is much higher in the peritoneal cavity when compared with those in other organs and compartments (e.g., blood). About 35–70% peritoneal B cells are B1 cells with a CD19^+^CD23^−^ phenotype (9), which are largely innate immune cells and are also self-renewing in the peritoneal cavity (10).

In recent years, the functions of peritoneal macrophages and B1 cells, as well as their interactions with each other, have been extensively explored (6, 9, 11). It has been reported that GATA6, a transcriptional factor, is characteristically expressed in LPMs but not in SPMs (12). Under the regulation of retinoic acid, GATA6 directs the expression and secretion of TGF-β from peritoneal macrophages, which in turn stimulates B1 cells to migrate into the intestine and secrete IgA (9). Through such a mechanism, the peritoneal macrophages and B1 cells play important roles in the maintenance of the gastrointestinal homeostasis (11).

Previously, we have reported that CPT-11, a chemotherapeutic agent, can eliminate resident peritoneal macrophages by inducing apoptosis (13). CPT-11 is a first-line drug used in clinic to treat colorectal cancer and small cell lung cancer (14). It exhibits cytotoxic effects on cancer cells by interfering with the DNA replication and transcription through inhibiting the activity of topoisomerase I and thus preventing the unwinding of DNA (15). However, CPT-11 itself does not specifically target cancer cells but is also toxic to normal tissues including the intestinal epithelium (16). It has been reported that CPT-11 can be converted into 7-ethyl-10-hydroxy-camptothecin (SN-38), a more potent inhibitor of topoisomerase when compared with CPT-11, by the hepatic carboxylesterase (17). SN-38 becomes the major metabolic product that is highly toxic to the intestine (18). Recent studies have identified the ileum, jejunum, and liver as the target tissues of CPT-11’s toxicity (19). Beyond its direct toxicity to the intestinal epithelial cells, it has also been shown to activate inflammasomes and IL-1β secretion through Janus N-terminal kinase and nuclear factor-κB signaling (20). Consequently, CPT-11 treatment may induce colitis as dextran sodium sulfate does (13, 20). In addition, CPT-11 may induce hyper-proliferation of mesenteric lymph node cells and hyper-production of inflammatory cytokines from them upon mitogen stimulation (21). It is known that chronic inflammation directs macrophages to polarize into M2 phenotype (22), which in turn benefits tumor growth (23–25). Indeed, CPT-11 treatment increases infiltration of macrophages and secretion of chemokines (including monocyte chemotactic protein 1) in tumor-bearing mice (26), when compared with vehicle treatment. Moreover, elimination of the peritoneal resident macrophages by CPT-11 (13) may impair the homeostasis of gastrointestinal immunity and thus weaken its actions against intestinal cancers.

Although we have demonstrated that CPT-11 can deplete the resident peritoneal macrophages (13), the dynamic details underlying this process remain unknown. For example, could the resident peritoneal macrophages and B1 cells recover from CPT-11 treatment by self-renewal? Does adoptive cell transfer benefit the recovery of these innate immune cells? In this study, we have addressed these issues by using a mouse model of CPT-11 treatment. Our results showed that the LPMs and B1 cells in the peritoneal exudate were almost fully depleted by CPT-11 within 1 week, but reappeared afterward. However, even until the end of the experiments (2 months after CPT-11 treatment), the restoring numbers of both LPMs and B1 cells were much fewer than those of vehicle-treated or untreated control groups. Adoptive transfer with syngeneic peritoneal exudate cells (PECs) accelerated the recovery of LPMs and B1 cells, but whether transfer with bone marrow cells (BMCs) had such an effect warranted longer observation. Our data revealed a prolonged deterioration of CPT-11 on the innate immune cells in the peritoneal cavity, which should be noted in the application of CPT-11 for cancer treatment in clinic.

## Materials and Methods

### Reagents and Antibodies

Dimethyl sulfoxide (D8418), Hoechst 33342 (B2261), Tween 80 (P8074), and paraformaldehyde (158127) were purchased from Sigma-Aldrich (St. Louis, MO, USA). Dulbecco’s modified Eagle’s medium (DMEM) with high glucose, penicillin, streptomycin, and fetal bovine serum (FBS) were products of ThermoFisher/Gibco (Carlsbad, CA, USA). CPT-11 (#HY-16562A) was purchased from MedChem Express (Princeton, NJ, USA), dissolved in ethanol before freshly diluted in phosphate-buffered saline (PBS) containing 2% Tween 80 in each experiment, and the final concentration of ethanol, never exceeded 5%. Fluorescence-labeled monoclonal antibodies CD11b-FITC (#11-0112), F4/80-PE (#12-4801), MHCII-APC (#17-5321), CD23-PE (#12-0232), CD19-eFluor660 (#50-0193), and CD11b-AlexaFluor488 (#53-0112) were obtained from eBioscience (San Diego, CA, USA); monoclonal anti-mouse F4/80-AlexaFluor 647 (#123121) were bought from BioLegend (San Diego, CA, USA). The rabbit monoclonal antibody against GATA6 (#5851) was purchased from Cell Signaling Technology (Danvers, MA, USA). CF568 goat-anti-rabbit IgG (H+L), highly cross-adsorbed (#201031) was obtained from Biotium (Hayward, CA, USA).

### Animals

Female C57BL/6 mice (6–8 weeks of age) were purchased from the Experimental Animal Center of Southern Medical University (Guangzhou, China). All animal experiments were performed in accordance with the guidelines for the care and use of animals approved by the Committee on the Ethics of Animals Experiments of Jinan University.

After 1 week of acclimatization, mice were randomly divided into groups with six mice in each group, and administered intraperitoneally (i.p.) with vehicle, CPT-11 (200 mg/kg body weight, at day 0) or untreated (control), respectively. The mice were sacrificed at day 3, day 7, day 14, day 28, and day 56, respectively.

In another experiment, mice were i.p. injected with CPT-11 (200 mg/kg body weight). After 7 days, the mice were injected (i.p.) with viable *Escherichia coli* bacteria (1 × 10^9^ CFU/mouse), which was freshly prepared as described previously (27). Their survival was observed and recorded every 6 h for four consecutive days.

Further in a separate experiment, mice were orally administered with CPT-11 (400 mg/kg body weight) once (at day 0) or twice (at day 0 and day 1), vehicle or left untreated. The mice were sacrificed at day 3, day 7, or day 14, respectively. The PECs were collected and analyzed as described below. The intestines and colons were isolated and fixed in 4% neutral formaldehyde. Paraffin slices of the tissues were stained with hematoxylin and eosin. Images were captured under a Zeiss Axio Observer D1 microscope armed with a color CCD (ZEISS).

### Isolation and Flow Cytometric Analysis of Mouse Peritoneal Cells

After indicated treatment and being sacrificed, each mouse was injected with 1.5 ml washing buffer (germ-free PBS containing 0.5 mM EDTA and 5% calf serum) into the peritoneal cavity and the peritoneal lavage fluid was collected. The PECs were washed once with PBS-F (PBS containing 0.1% NaN_3_ and 3% FBS) by centrifugation at 300 × *g* for 5 min, and then stained with FITC labeled anti-CD11b, PE labeled anti-F4/80, and APC labeled anti-MHCII, or PE-conjugated anti-CD23 and eFluor660-conjugated anti-CD19 monoclonal antibodies at 4°C for 30 min. Red blood cells, if there were, were lysed with ACK lysis buffer (155 mM NH_4_Cl, 10 mM KHCO_3_, and 0.1 mM EDTA). After washing once with PBS-F, cells were fixed with 4% paraformaldehyde in PBS and then analyzed on a flow cytometer (FACSCalibur; Becton Dickinson). Data were acquired and analyzed by using the CELLQuest software (Becton Dickinson).

### Cell Culture and Fluorescence Microscopy

Immunofluorescence analysis was performed essentially as previously described (28). Briefly, PECs were collected by centrifugation at 300 × *g* for 5 min and re-suspended in complete DMEM medium containing 10% FBS, 100 U/ml penicillin, and 100 µg/ml streptomycin. Then the cells were seeded in glass-bottomed dishes (5 × 10^5^ cells/dish). After 2-h incubation at 37°C in a humidified incubator of 5% CO_2_, unattached cells were discarded. After washed with PBS, the adherent macrophages were fixed in 4% paraformaldehyde for 15 min, and permeabilized with 2 ml cold methanol (−20°C) for 10 min. Then the cells were incubated with AlexaFluor488-CD11b (1:80), AlexaFluor647-F4/80 (1:100), and GATA6 (1:300) antibodies overnight, followed by being stained with CF568-conjugated goat-anti-rabbit IgG (1:750) for 1 h. The nuclei were revealed by Hoechst 33342 staining (5 µg/ml in PBS) for 10 min. The cells were observed by the Zeiss Axio Observer D1 microscope with a Zeiss LD Plan-Neofluar 100×/0.6 Korr M27 objective len (Carl Zeiss MicroImaging GmbH, Göttingen, Germany). Fluorescence images were captured and analyzed by the Zeiss ZEN software.

### Syngeneic Adoptive Transfer with Peritoneal Exuded Cells and BMCs

Peritoneal exudate cells were collected with 2 ml washing buffer (germ-free PBS containing 0.5 mM EDTA and 5% calf serum), centrifuged at 300 × *g* for 5 min at 4°C, and then washed once with 2 ml cold PBS. The cells were re-suspended in PBS at 2 × 10^6^ cells/ml, being ready for transplantation.

In preparing BMSs, the bone marrow from hind femora was flushed out with 10 ml of sterile cold PBS and the cells were collected by centrifugation at 300 × *g* for 5 min at 4°C. Red blood cells were lysed in ACK lysis buffer for 10 min at 37°C, stopped with cold DMEM containing 10% FBS, 100 U/ml penicillin, and 100 µg/ml streptomycin. The cells were washed once with 2–3 ml cold PBS, and then re-suspended in PBS to 2 × 10^7^ cells/ml. All procedures were performed in an aseptic surrounding.

Mice were i.p. treated with CPT-11 for 7 days, and then 0.5 ml of peritoneal exuded cells (1 × 10^6^ cells) or BMCs (1 × 10^7^ cells) were injected into the peritoneal cavity. After additional 21 days, the mice were sacrificed and their PECs were collected and analyzed by flow cytometry and immunofluorescence microscopy, respectively.

### Statistical Analysis

All experiments were performed three times independently, with one representative experiment shown. Data were expressed as mean ± SD. Statistical analysis was performed using GraphPad Prism 5.0 (GraphPad Software Inc., San Diego, CA, USA). One-way analysis of variance followed by Tukey *post hoc* test and unpaired Student’s *t*-test were used to analyze the statistical significance among multiple groups and between two groups, respectively. Kaplan–Meier survival curves were adopted for analysis of mice survival, and the statistical difference between two groups was determined using the log-rank (Mantel–Cox) test. *P*-values < 0.05 were considered statistically significant.

## Results

### The Rapid Impairment of Resident Peritoneal Macrophages after i.p. CPT-11 Treatment Required a Prolonged Recovery Period

**Figure 1:**
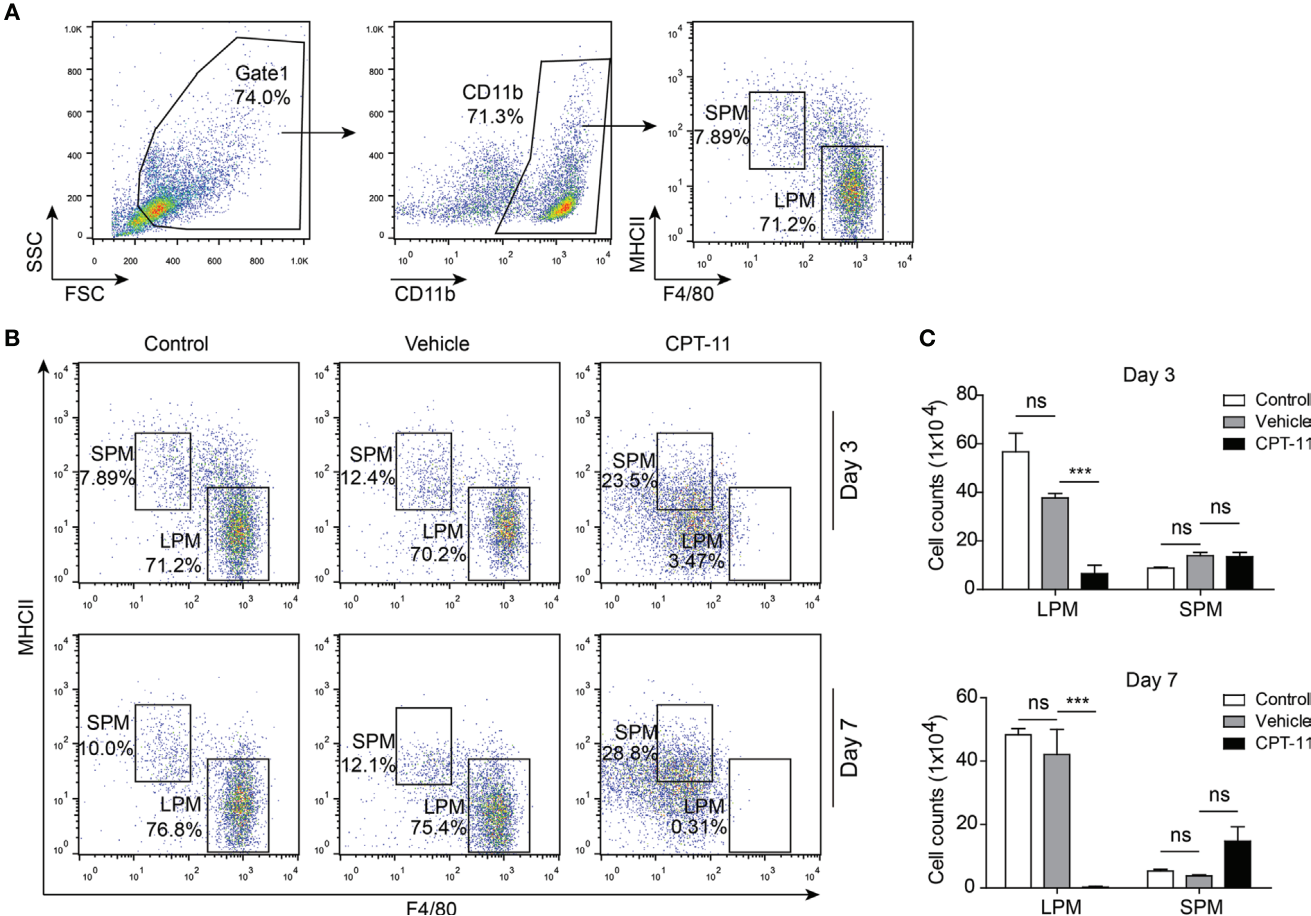
CPT-11 treatment rapidly reduced the numbers of peritoneal resident macrophages. **(A)** CD11b-positive cells were gated and the peritoneal macrophages, including large peritoneal macrophages (LPMs) and small peritoneal macrophages (SPMs), were determined by their cell surface expression of F4/80 and MHC II using fluorescent-conjugated antibodies together with flow cytometry. A representative set of dot-plots of control group (day 3) is presented. **(B)** The composition of LPMs (F4/80^hi^MHC-II^low^) and SPMs (F4/80^low^MHC-II^hi^) after CPT-11 treatment were analyzed using the methods as shown in **(A)**. The results showed that CPT-11 treatment reduced the ratios of LPMs at both day 3 and day 7. **(C)** The cell numbers of LPMs and SPMs were calculated by their relative ratios times the total peritoneal exudate cell numbers (determined by a hemocytometer), respectively. The results show that the LPM cell numbers were also greatly reduced by CPT-11 treatment. *n* = 6; ***P < 0.01; ns, not significant.

The dynamic process of CPT-11-induced damage to the peritoneal macrophages was assessed in mice administered with CPT-11 (i.p.). After CPT-11 treatment, the phenotypes of the PECs from the mice of each group were analyzed every week by flow cytometry. The LPMs (F4/80^hi^MHC-II^low^) and SPMs (F4/80^low^MHC-II^hi^) were gated and their ratios and numbers were determined, respectively (Figure 1A). The results showed that neither the ratios nor the numbers of LPMs and SPMs were significantly changed by vehicle when compared with untreated control. In contrast, the ratios and numbers of LPMs were greatly decreased by CPT-11 treatment. Notably, LPMs in CPT-11-treated group were hardly detectable at day 7. However, the numbers of SPMs were not significantly changed by CPT-11, despite their increased ratios within the CD11b^+^ population (Figures 1B,C). Together with previous studies (13), these results indicated that the resident peritoneal macrophages were rapidly depleted by i.p. CPT-11 treatment.

**Figure 2:**
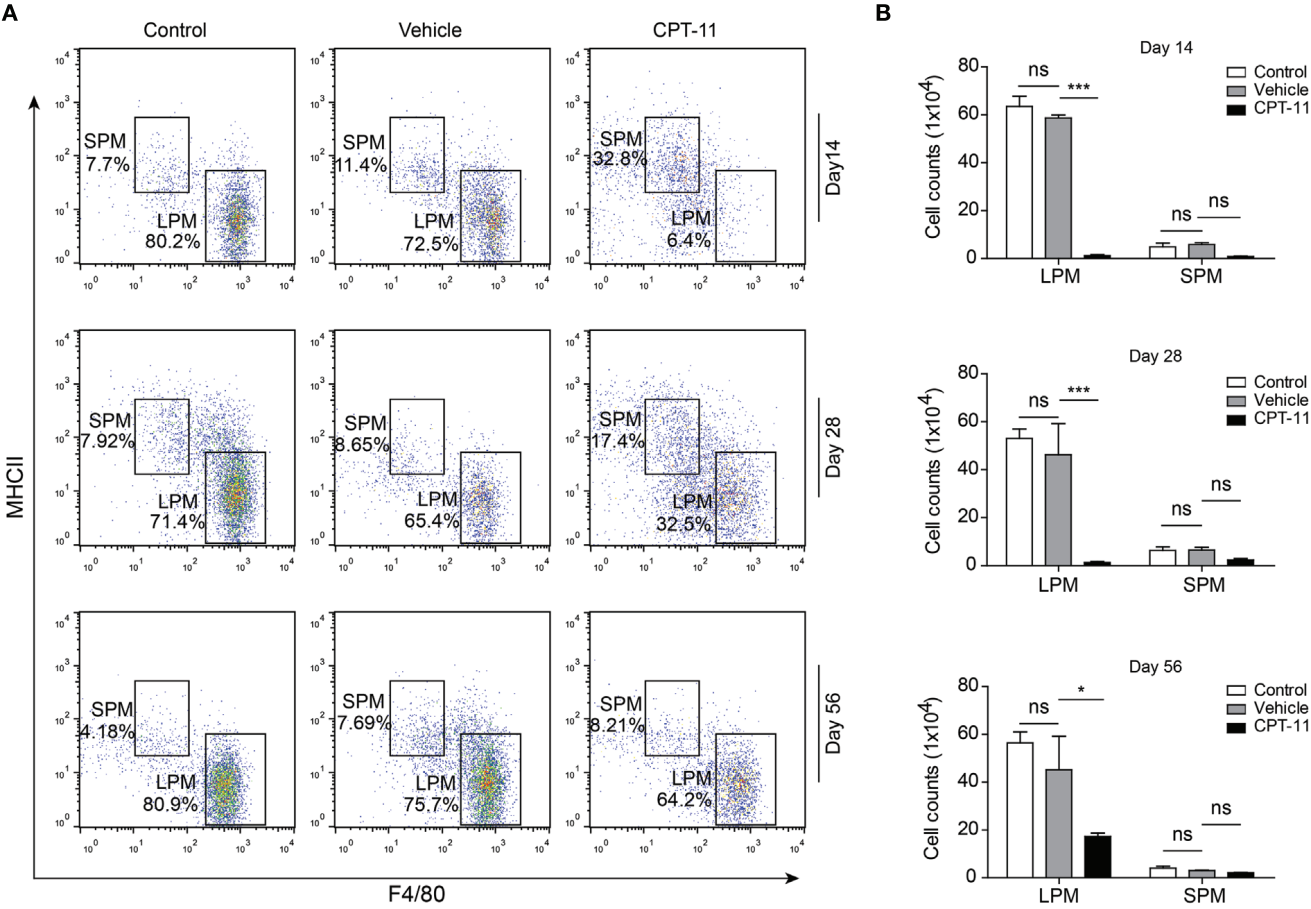
The cell numbers of large peritoneal macrophages (LPMs) began to recover 2 weeks after CPT-11 treatment. **(A)** C57BL/6 mice (six mice per group) were injected intraperitoneally with CPT-11 at a dose of 200 mg/kg body weight. The composition of LPMs and small peritoneal macrophages (SPMs) after CPT-11 treatment was analyzed by flow cytometry using fluorescent-conjugated antibodies against CD11b, F4/80, and MHC II. **(B)** Cell numbers of the LPM and SPM populations were calculated as Figure 1. *n* = 6; *P < 0.05; ***P < 0.001; ns, not significant.

Next, we sought to explore the recovery dynamic process of resident peritoneal macrophages after CPT-11 treatment. Flow cytometry revealed that LPMs were detected again at day 14 and from then on their numbers were gradually increased (Figure 2A). However, although the ratios of LPMs in CPT-11 groups were time-dependently increased, their cell numbers were still much lower than those of vehicle-treated control group. The cell numbers of LPMs in CPT-11-treated group were ~30% of that in vehicle-treated control group even after 56 days. Again, the numbers of SPMs (F4/80^low^MHC-II^hi^) were barely influenced by CPT-11 treatment (Figures 2A,B). These results indicated that CPT-11 administration had prolonged deleterious effects on the resident peritoneal macrophages.

### The Dynamic Process of GATA6 Expression in Peritoneal Macrophages after i.p. CPT-11 Treatment

**Figure 3:**
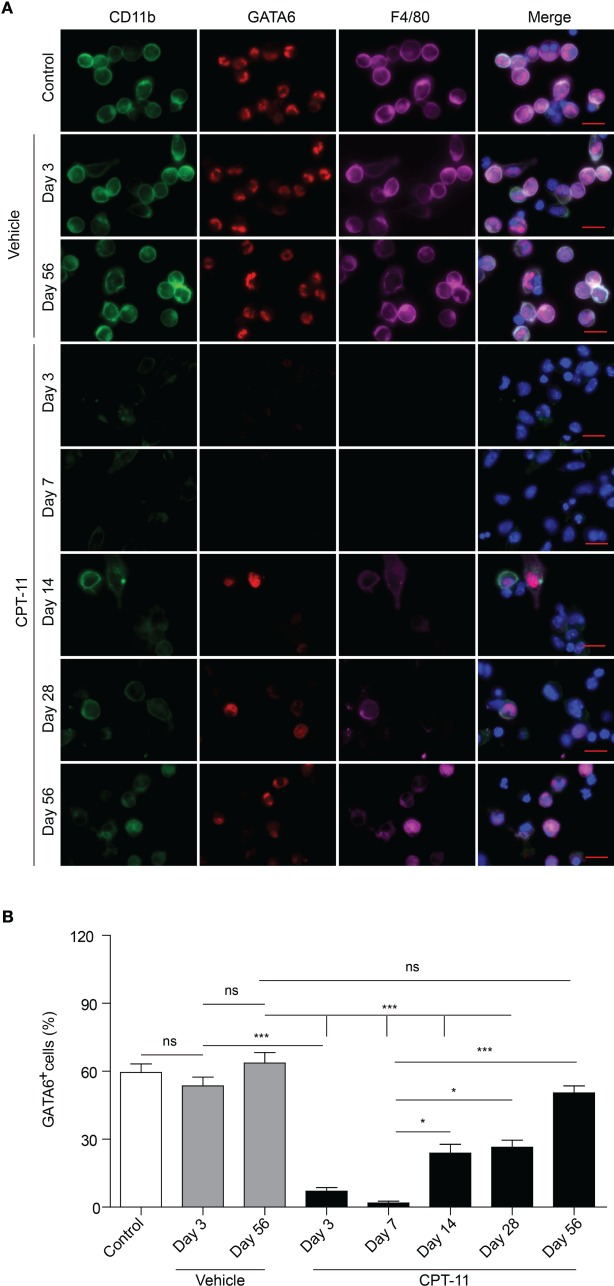
Immunofluorescent observation of the expression of CD11b, F4/80, and GATA6 in peritoneal macrophages after CPT-11 treatment. **(A)** C57BL/6 mice (six mice per group) were injected intraperitoneally with CPT-11 at a dose of 200 mg/kg body weight. Peritoneal exudate cells were collected and planted in dishes (5 × 10^5^/dish) *ex vivo* while non-adherent cells were discarded 2 h later. Then the attached cells (macrophages) were stained with antibodies against CD11b (green), F4/80 (magenta), and GATA6 (red). The cells were observed under a Zeiss fluorescent microscope, with the nuclei (blue) being revealed by Hoechst 33342, and the fluorescence images were captured and analyzed by the Zeiss ZEN software. Scale bar: 10 mm. **(B)** The ratios of GATA6^+^ cells were calculated using the Zeiss ZEN software. *n* = 6; *P < 0.05; ***P < 0.001; ns, not significant.

To verify the flow cytometric data showing that LPMs can be recovered from CPT-11 treatment, we next used immunofluorescent staining to detect the expression of GATA6, a functional marker of LPMs (9), in PECs. The PECs were cultured *ex vivo*. The macrophages among them were attached to the culture dish thus being selected by discarding the non-adherent cells, and were further observed by immunofluorescence microscopy using antibodies against CD11b, F4/80, and GATA6. As shown in Figure 3A, the expression of GATA6 was not significantly influenced by vehicle treatment; but after CPT-11 treatment, the cells expressing GATA6 were drastically decreased at day 3 and were barely detectable at day 7, indicative of almost complete loss of functional LPMs during these early stages. Supporting this, the expression of CD11b (a marker of myeloid cells) and F4/80 (a marker for mature macrophages) was also suppressed by CPT-11 treatment (Figure 3A). At day 14, however, GATA6 expression was detected in a minor population of the peritoneal macrophages and since then the GATA6-expressing cells were gradually increased. Concomitant with the re-expression of GATA6, both CD11b and F4/80 expressions were gradually reappeared, indicative of the recovery of mature macrophages.

Quantification of immunofluorescent observation showed that the percentages of GATA6^+^ macrophages were rapidly decreased within 1 week by CPT-11 treatment but slowly recovered after day 14 (Figure 3B), which was consistent with the flow cytometric analysis of LPMs (Figures 1B and 2B). Notably, although the percentage of GATA6^+^ LPMs in CPT-11-treated group (day 56) was comparable with the counterpart of corresponding vehicle-treated control group, their absolute cell counts (~7.5 × 10^5^ PECs) were much fewer than the latter group (~1.5 × 10^6^ PECs). These results also showed that CPT-11 administration induced a rapid loss but a very slow recovery of the functional LPMs with GATA6 expression, indicating a prolonged deterioration of these innate immune cells in the peritoneal cavity of mice.

### The Dynamic Process of Peritoneal B Cells after i.p. CPT-11 Treatment

**Figure 4:**
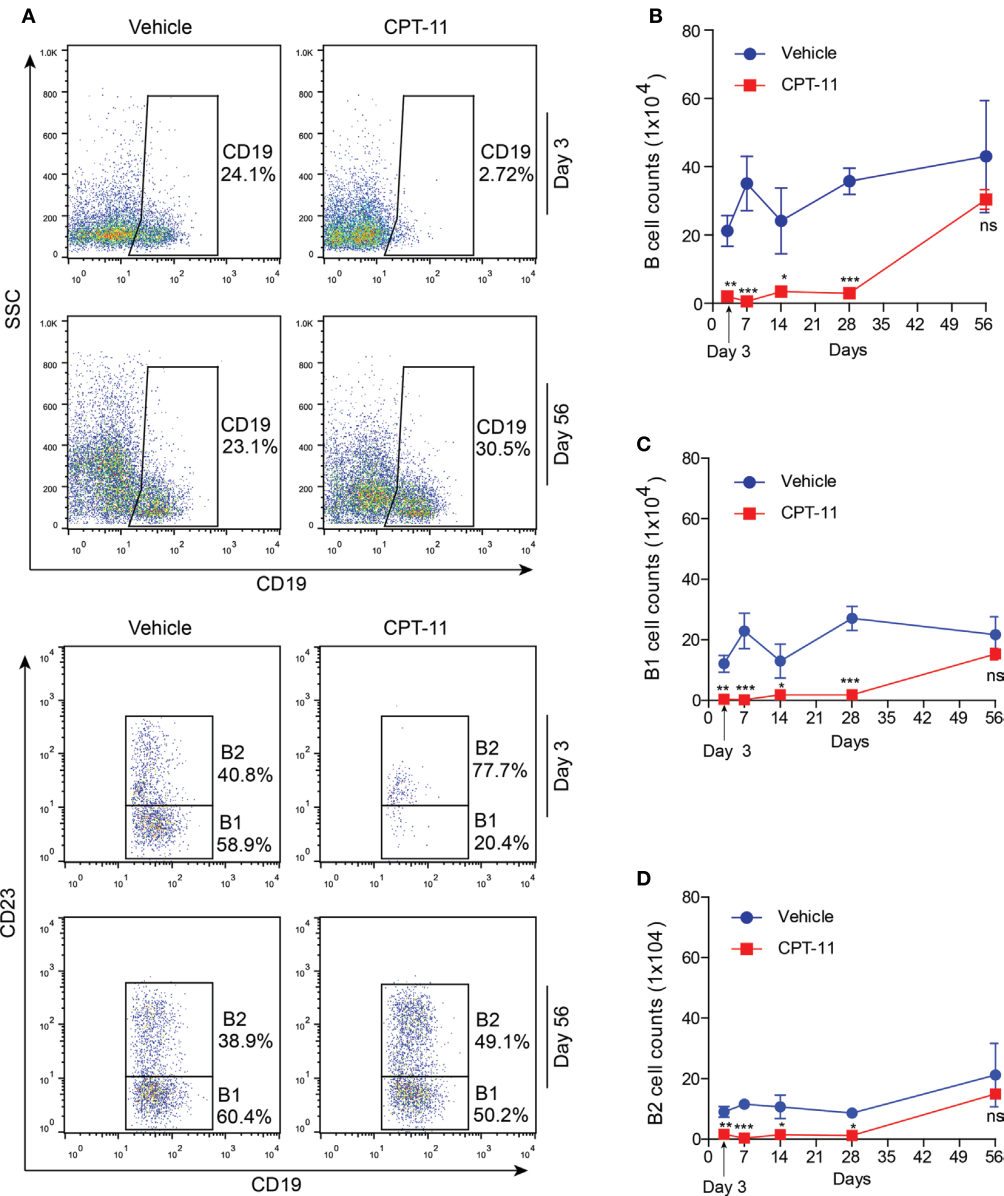
The dynamic process of peritoneal B cells after CPT-11 treatment. **(A)** C57BL/6 mice were injected intraperitoneally with CPT-11 at a dose of 200 mg/kg body weight. The peritoneal exudate cells (PECs) were stained with antibodies against CD19 and CD23 and then analyzed by flow cytometry. B1 cells were identified as CD19^+^CD23^−^ cells and B2 as CD19^+^CD23^+^ ones. **(B–D)** The numbers of total B (CD19^+^) **(B)**, B1 (CD19^+^CD23^−^) **(C)**, and B2 (CD19^+^CD23^+^) **(D)** cells were calculated by their percentages times the total PEC numbers (determined by a hemocytometer), respectively. *n* = 6; *P < 0.05; **P < 0.01; ***P < 0.001; ns, not significant.

Apart from resident macrophages, B cells, particularly B1 cells constitute another major population of the PECs. Thus, we next assayed the dynamic process of these cells after CPT-11 treatment. The PECs were stained with CD19 and CD23 antibodies, and analyzed by flow cytometry. Among them, B1 cells were identified as CD19^+^CD23^−^ cells while B2 cells as CD19^+^CD23^+^ ones. The results showed that the numbers of B cells (CD19^+^) were not significantly changed by vehicle treatment when compared with untreated control (data not shown), but were greatly reduced by CPT-11. Three days after CPT-11 treatment, only ~3% of the PECs were B cells (Figures 4A,B). Consequently, the numbers of both B1 and B2 cells were reduced by CPT-11 administration (Figures 4C,D). Interestingly, the numbers of total B cells as well as B1 and B2 cells were restored at day 56, comparable with that of vehicle-treated group (Figure 4). These results demonstrated that the peritoneal B cells were quickly impaired by CPT-11 treatment but were recoverable within 2 months.

### Adoptive Transfer of Syngeneic PECs Accelerated the Recovery Processes of LPMs and B1 Cells in the i.p. CPT-11-Treated Mice

**Figure 5:**
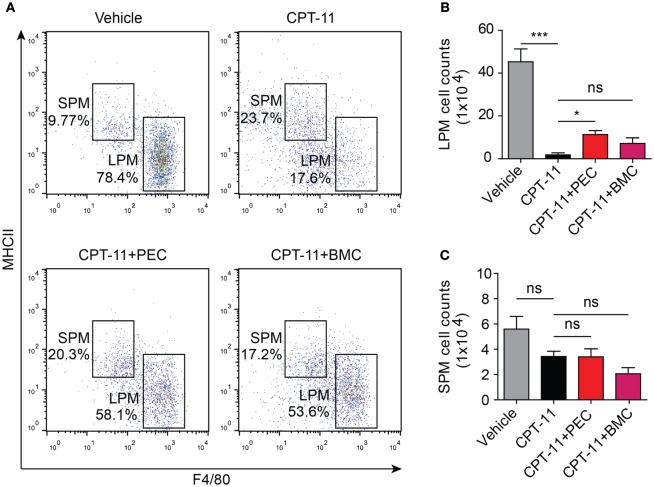
Syngeneic adoptive transfer with peritoneal exudate cells (PECs) rescued the resident peritoneal macrophages after CPT-11 treatment. C57BL/6 mice were intraperitoneally injected with CPT-11 (200 mg/kg body weight, in phosphate-buffered saline containing 2% Tween-80) or vehicle. Seven days later, the CPT-11 group mice were transferred with syngeneic PECs (1 × 10^6^/mouse) or bone marrow cells (BMCs, 1 × 10^7^/mouse). The mice were cultivated for 21 days more, and then their PECs were analyzed by flow cytometry after being stained with fluorescent-conjugated antibodies against CD11b, F4/80, and MHC II. **(A)** A representative set of flow cytometric dot-plots was presented. **(B,C)** The numbers of large peritoneal macrophages (LPMs) and small peritoneal macrophages (SPMs) were calculated by their percentages times the total peritoneal cell numbers (determined by a hemocytometer), respectively. *n* = 6; *P < 0.05; ***P < 0.001; ns, not significant.

As CPT-11 treatment rapidly reduced the numbers of both LPMs and B cells within 1 week but the recovery processes were slow, we next sought to explore whether adoptive cell transfer accelerated the recovery processes. Mice were received syngeneic PECs and BMCs at day 7 after CPT-11 treatment. The phenotypes of the PECs were analyzed 21 days after the adoptive transfer. The results showed that the adoptive transfer with PECs significantly increased the ratios and numbers of LPMs (F4/80^hi^MHC-II^low^) in CPT-11-treated mice when compared with those of CPT-11-treated control group without adoptive transfer, although these values were still lower than those of vehicle-treated control group. The adoptive transfer with BMCs also slightly increased the numbers of LPMs when compared with those of CPT-11-treated group without adoptive transfer, but the differences were not statistically significant (Figures 5A,B). Besides, the ratios and numbers of SPMs were not significantly changed by the adoptive PECs or BMCs (Figures 5A,C).

**Figure 6:**
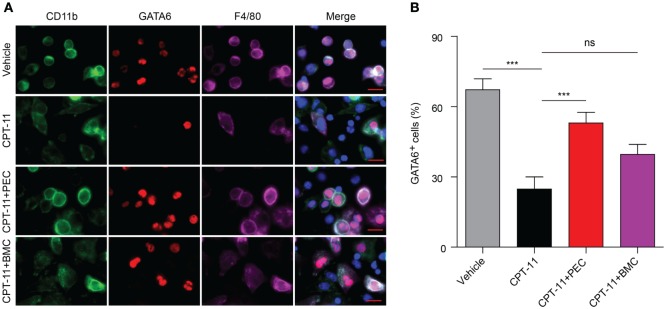
Immunofluorescence observation of the expression of GATA6 in the peritoneal macrophages after CPT-11 treatment and syngeneic adoptive transfer with peritoneal exudate cells (PECs) and bone marrow cells (BMCs). C57BL/6 mice were intraperitoneally injected with CPT-11 (200 mg/kg body weight, in phosphate-buffered saline containing 2% Tween-80) or vehicle. Seven days later, the CPT-11-treated mice were transferred with syngeneic PECs (1 × 10^6^/mouse) or BMCs (1 × 10^7^/mouse). The mice were bred for additional 21 days, and then their PECs were collected. The expression of CD11b (green), F4/80 (magenta), and GATA6 (red) was observed by immunofluorescence microscopy with the nuclei (blue) being revealed by Hoechst 33342 staining. **(A)** A representative set of immunofluorescence images. Scale bar: 10 mm. **(B)** Statistical analysis of the percentages of CD11b^+^GATA6^+^ macrophages. *n* = 6; ***P < 0.001; ns, not significant.

Consistent with the flow cytometry analysis, immunofluorescence microscopy also revealed that GATA6 expression in the peritoneal macrophages was greatly suppressed by CPT-11 treatment, but transfer of PECs into the peritoneal cavity significantly increased the percentage of GATA6^+^ macrophages when compared with CPT-11-treated control group (Figures 6A,B). The adoptive transfer of BMCs also increased GATA6 expression, although this was not statistically significant (Figure 6B).

**Figure 7:**
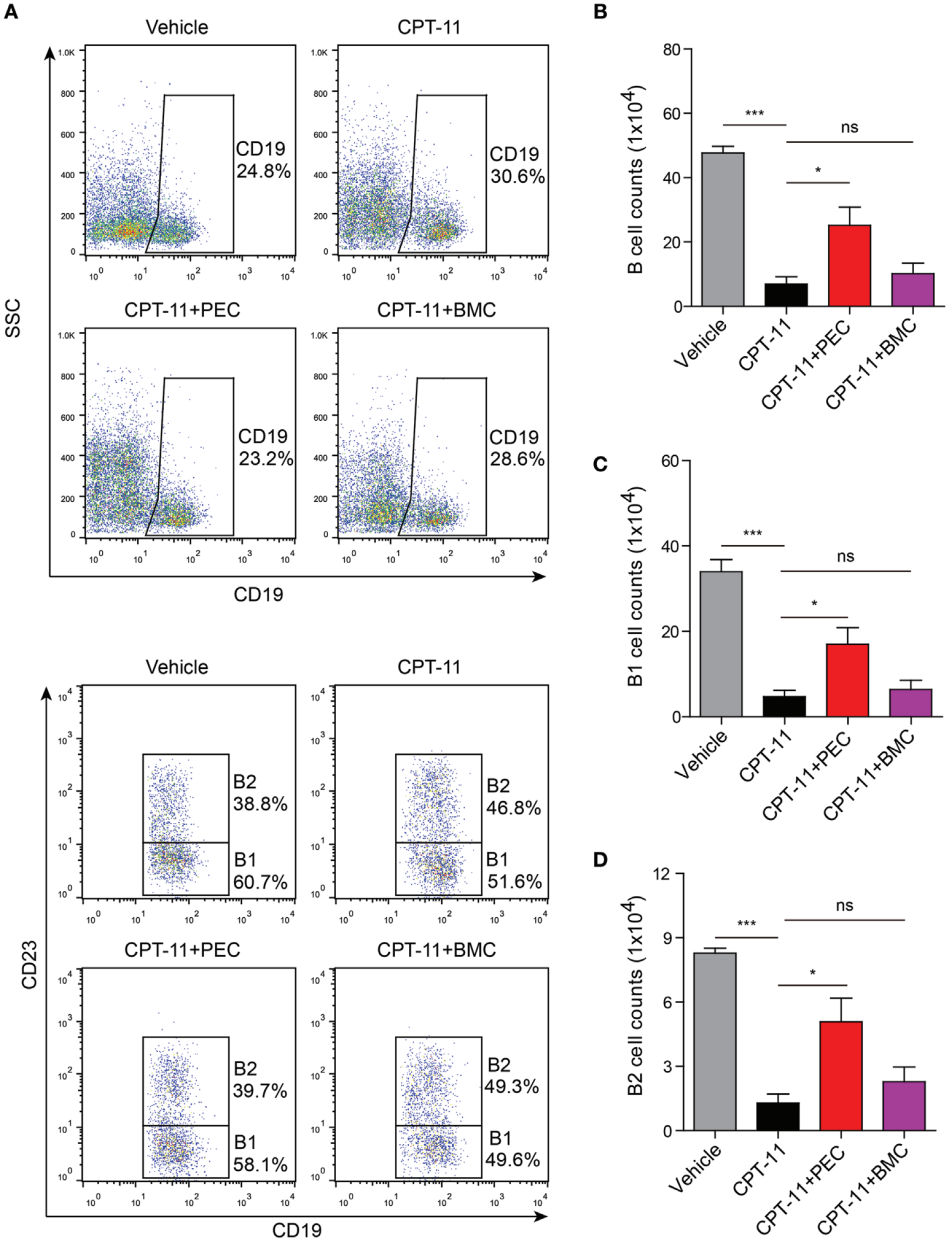
Analysis of the B cell populations by flow cytometry after the syngeneic adoptive transfer with peritoneal exudate cells and bone marrow cells. C57BL/6 mice were treated as described in Figure 5. **(A)** The B cell populations (including B1 and B2 subpopulations) in the peritoneal cavity were analyzed by flow cytometry. B cell populations were gated as CD19^+^ cells, among which B1 cells were CD19^+^CD23^−^ ones while B2 cells were CD19^+^CD23^+^ ones. **(B, C)** and **(D)** Statistical analysis of the B **(B)**, B1 **(C)**, and B2 **(D)** cell counts in each group. *n* = 6; *P < 0.05; ***P < 0.001; ns, not significant.

Similarly, the adoptive transfer of PECs also rescued the B cell population (including B1 and B2 subpopulations) in the peritoneal cavity of the CPT-11-treated mice. However, transfer of BMCs was unlikely to help the recovery of B cell counts that had been decreased by CPT-11 treatment (Figure 7). Together, the adoptive transfer of PECs, instead of BMCs, promoted the recovery of resident peritoneal macrophages and B cells in CPT-11-treated mice.

### i.p. Treatment with CPT-11 Reduced Mouse Survival Rate against Bacterial Infection

**Figure 8:**
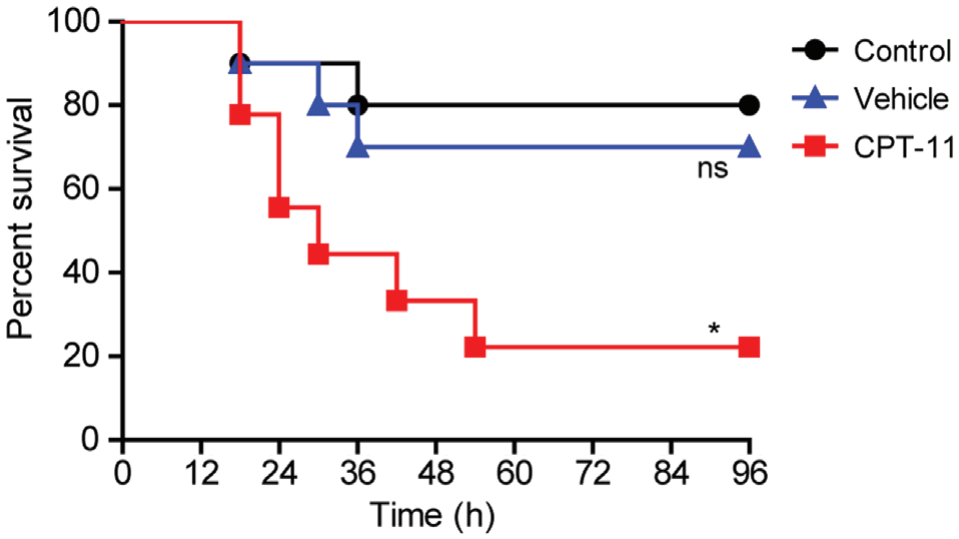
Intraperitoneal (i.p.) treatment with CPT-11 reduced mouse survival rate against bacterial infection. C57BL/6 mice (female, 6–8 weeks old) were first i.p. injected with CPT-11 (200 mg/kg body weight at day 0). Food and water were freely available. At day 7, viable *Escherichia coli* at a dose of 1 × 10^9^ CFU/mouse was injected into the peritoneal cavity of each mice (including control and vehicle groups). The mice were observed every 6 h for four consecutive days. Kaplan–Meier survival curves were used to analyze the data. The significance was determined by the log-rank (Mantel–Cox) test. *n* = 10; *P < 0.05; ns, not significant vs control.

We next explored the functional consequence of the impairment of resident peritoneal macrophages in CPT-11-administered mice. The mice were first i.p. injected with CPT-11 and 7 days later their peritoneal cavity were infected with viable *E. coli*. As shown in Figure 8, CPT-11 treatment significantly reduced the mouse survival rate when compared with that of vehicle-treated or untreated control groups. This suggested that the reduction of peritoneal macrophages and B cells upon CPT-11 treatment had significantly weakened the innate immunity against bacterial infection.

### Oral Administration of CPT-11 Induced a Delayed Toxicity to the Peritoneal Macrophages When Compared with i.p. Treatment

Oral administration of CPT-11 has been shown to be active against tumors in nude mice (29), but most of the animal experiments using CPT-11 were performed by i.p. injection according to the protocol of Battelle Columbus laboratories, which has displayed similar pharmacokinetics to those by intravenous (i.v.) administration (29, 30). In this study, we investigated whether CPT-11 by oral intake also changed the components of the PECs and induced intestinal inflammation in mice as it does by i.p. injection (20, 31). Different from i.p. route, oral administration of CPT-11 twice (at day 0 and day 1) at the dose of 400 mg/kg in C57BL/6 mice did not significantly reduced the percentages of peritoneal macrophages (LPMs and SPMs) (Figure S1 in Supplementary Material) and of B cells (Figure S2 in Supplementary Material) at day 3. Nor did it changed the expression of GATA6 in the peritoneal macrophages (Figure S3 in Supplementary Material). However, the total peritoneal exude cells reduced about one-third (from ~1.5 × 10^6^ to ~9.5 × 10^5^). Moreover, oral CPT-11 administration induced severe epithelial vacuolation in the full-length of the intestines (Figure S4 in Supplementary Material) and the animals began to die from day 4 and on (data not shown).

In another experiment, the drug was administered orally at the same dose only once (at day 0). The total number of the PECs in the CPT-11 group were decreased to ~9 × 10^5^ (~1/3 reduction when compared with vehicle-treated or untreated control) at both day 7 and day 14. Again, oral CPT-11 treatment did not change the percentages of peritoneal macrophages at day 7 (data not shown), but those of LPMs were decreased by CPT-11 at day 14 (Figure S5 in Supplementary Material). The ratios and numbers of peritoneal B cells were unchanged until day 14, except that the percentages of B2 cells seemed increased by CPT-11 at day 14 (Figure S6 in Supplementary Material). However, the expression of GATA6 in the peritoneal macrophages was unchanged (Figure S7 in Supplementary Material). Therefore, in addition to induction of exaggerated intestinal inflammation, oral CPT-11 administration showed a delayed cytotoxicity to the peritoneal macrophages when compared with i.p. CPT-11 treatment.

## Discussion

CPT-11 is used as a first-line drug for colorectal cancer treatment in clinic. However, there are reports indicating that CPT-11 is also toxic to the intestinal epithelium and peritoneal innate immune cells (13, 16, 32), while i.v. CPT-11 usually induces vomiting and neutropenia in patients (33).

In this study, we demonstrated the prolonged influences of CPT-11 on mouse resident peritoneal macrophages and B1 cells. After CPT-11 treatment, the whole populations of F4/80^hi^GATA6^+^ macrophages (i.e., LPMs) and CD19^+^CD23^−^ B1 cells were almost completely depleted. LPMs were hardly detectable at day 7 after CPT-11 treatment, but reappeared at day 14. However, the recovery process was slow in that the peritoneal macrophages were not completely recovered within 2 months.

Resident peritoneal macrophages are self-renewing from their progenitors in the omentum (3, 34). If without the existing progenitors, the self-renewal of resident peritoneal macrophages should be hampered. Thus, the slow recovery process of LPMs was likely due to severe damage to their self-renewing progenitors after CPT-11 treatment. A minor remnant of these self-renewing progenitors may survive CPT-11 treatment and slowly replenish the LPMs. On the other hand, it has been demonstrated that bone marrow-derived (circulating) monocytes can enter into the peritoneal cavity, becoming F4/80^low^MHCII*^+^* macrophages (phenocopying the SPMs) (7), and the latter can be further developed into F4/80^hi^GATA6^+^ macrophages (5). Although LPMs are self-renewing in the steady state, they can be reconstituted by differentiation from BMC precursors after irradiation in chimeric mice (35). Notably, only ~20% of the F4/80^hi^ macrophages can be reconstituted from the donor bone marrow by 36 weeks (5). Consistent with the slow reconstitution of macrophages in that report, our data revealed that the recovery process of F4/80^hi^GATA6^+^ macrophages in CPT-11-treated mice was also slow as only one-third of normal cell numbers were recovered even after 2 months of CPT-11 treatment. However, it is still unclear whether the reappeared LPMs were developed from the circulating monocytes or the remnant of their self-renewing progenitors in the omentum in the context of our experimental setting. Further study is required to clarify this issue.

Many studies have focused on the undesirable toxicity of CPT-11 on normal tissues in humans beyond its cytotoxic effects on cancer cells (36). It has been shown that CPT-11 can induce diarrhea and vomiting (37). Although it is unknown whether depletion of the peritoneal macrophages by CPT-11 contributed to these side effects, the toxicity of CPT-11 on both resident peritoneal macrophages and B1 cells deserves thorough research due to their fundamental functions in maintaining homeostasis of the intestine. It has been reported that both of the SPMs and LPMs can engulf invaded pathogens (6), but it was the LPMs that engulfed bacteria immediately (within 0.5 h) (28). Notably, once the liver (a peritoneal organ) is damaged, the resident peritoneal macrophages (LPMs) quickly move to the wound and take part in the repair process (38), suggesting that they may also repair other peritoneal organs including the intestine when it is damaged. Besides, resident peritoneal macrophages can interact with B1 cells and promote the latter to migrate into the intestine and secrete IgA (9). Dimeric IgA, which is also known as secretory IgA, can survive in the harsh intestinal tract environment and effectively prevent pathogens from invading into the intestinal epithelium (39). Therefore, resident peritoneal macrophages and B1 cells are responsible for the maintenance of gastrointestinal innate immune cells. CPT-11-induced impairment of the peritoneal innate immune cells may weaken the first line of defense against infections. Indeed, the i.p. treatment with CPT-11 had greatly reduced the survival rate of mice upon bacterial infection. In this sense, the toxicity of CPT-11 on these innate immune cells may do great harm to the homeostasis of the gastrointestinal innate immunity.

CPT-11 is used in humans by i.v. injection. While in mouse experiments, CPT-11 is usually administered by i.p. injection according to the protocol of Battelle Columbus laboratories (29, 30). Although i.p. administration of CPT-11 is regarded as a usual route in mice to mimic the i.v. injection in humans (29, 30), oral gavage has also been used in animal studies (29). Thus, it is worth to know whether oral administration of CPT-11 also impairs the peritoneal innate immunity. To address this issue, mice were orally administered with CPT-11 once (at day 0) or twice (at day 0 and day 1). Interestingly, the total PECs were reduced about one-third as detected at day 3 (CPT-11 once or twice), but were not further reduced at day 7 or day 14 (CPT-11 once). When compared with the i.p. treatments, oral administration induced a delayed toxicity to the peritoneal macrophages as the percentages and numbers of LPMs were significantly reduced at day 14. Notably, oral administration twice with CPT-11-induced severe intestinal epithelial vacuolation and the mice began to die from day 4 (so only the data of day 3 were shown). Thus, oral CPT-11 treatment seemed less toxic to the peritoneal cells, but had elicited severe intestinal inflammation, which may have hampered its potential use by oral route.

As peritoneal innate immune cells play a fundamental role in gastrointestinal homeostasis, it is worth to explore whether adoptive cell transfer could accelerate the recovery of the peritoneal innate immune cells after CPT-11 administration. Consistent with previous studies that the F4/80^hi^ peritoneal macrophages can be reconstituted by circulating monocytes (7) or donor bone marrow (5), our current studies showed that the recovery of F4/80^hi^ peritoneal macrophages and B1 cells was accelerated by adoptive transfer of syngeneic PECs. Although it remains to be investigated whether such cell transplantation could attenuate the adverse toxicity of CPT-11, the adoptive transfer of PECs may be of translational significance for cancer treatment using CPT-11. Both the resting peritoneal macrophages and B1 cells are long-lived cells and can be isolated, if necessary, from cancer patients and be cryo-preserved for later use. Being revived *in vitro*, the peritoneal macrophages or B1 cells may be transplanted back into the peritoneal cavity after CPT-11 chemotherapy to accelerate the recovery of the peritoneal macrophages and B1 cells.

It should be noted that being different from the transplantation of PECs, the adoptive transfer of BMCs only slightly but not significantly increased the numbers of LPMs and B1 cells. This may be because the transition of donor BMCs into LPM-like macrophages or B1-like cells in the peritoneal cavity needs a longer period, as has been suggested previously (5, 40). Therefore, longer observation is warranted to learn whether autologous BMCs can be developed into LPMs and B1 cells in the peritoneal cavity. Another possibility is that adult BMCs were recruited in this study. Previous reports have suggested that aged BMCs have lost their ability to differentiate into peritoneal B1 cells (40).

In summary, i.p. CPT-11 treatment induced the depletion of resident peritoneal macrophages (LPMs) and B cells (including B1 and B2 cells), but the recovery processes of these cells were slow. Adoptive transfer with syngeneic PECs into the CPT-11-treated peritoneal cavities accelerated the recovery of these innate immune cells. Although further functional studies are needed, our studies highlight the potential of adoptive cell transfer as a way to counteract the adverse effects of this chemotherapeutic agent.

## Ethics Statement

Female C57BL/6 mice (6–8 weeks of age) were purchased from the Experimental Animal Center of Southern Medical University (Guangzhou, China). All animal experiments were performed in accordance with the guidelines for the care and use of animals approved by the Committee on the Ethics of Animals Experiments of Jinan University.

## Author Contributions

W-JB, L-HX, C-CZ, BH, and ZH conducted animal studies; W-JB, C-GL, L-HX, and Q-ZZ performed flow cytometry and immunofluorescence assays; W-JB and C-GL analyzed the data; D-YO and X-HH supervised the study; and D-YO, X-HH, and W-JB wrote the paper.

## Conflict of Interest Statement

The authors declare that the research was conducted in the absence of any commercial or financial relationships that could be construed as a potential conflict of interest.
