# Supplementary material for: Prolonged Deleterious Influences of Chemotherapeutic Agent CPT-11 on Resident Peritoneal Macrophages and B1 Cells
Source: Front Immunol. 2018 Jan 5;8:1919. doi: 10.3389/fimmu.2017.01919 (PMC5760539; doi:10.3389/fimmu.2017.01919)
Supplement: Supplementary file 1 [file Data_Sheet_1.doc]

**Supplementary Materials**

**Prolonged deleterious influences of chemotherapeutic agent CPT-11 on resident peritoneal macrophages and B1 cells**

Wen-Jing Bai, Chen-Guang Li, Cheng-Cheng Zhang, Li-Hui Xu, Qiong-Zhen Zeng, Bo Hu, Zhou Hong, Xian-Hui He, and Dong-Yun Ouyang


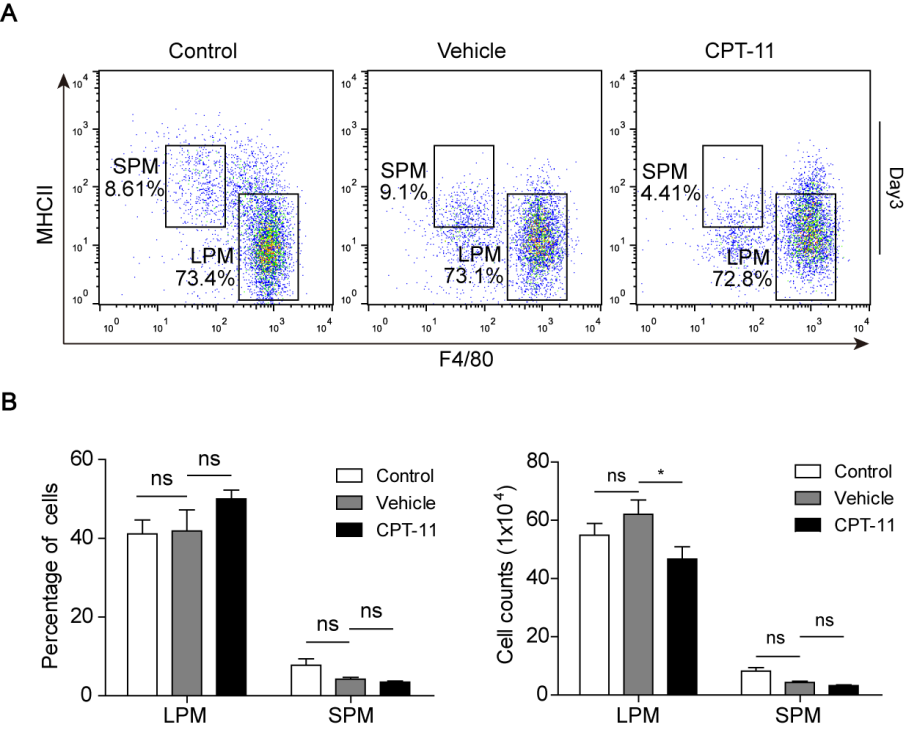


**Figure S1 ׀ Peritoneal macrophages in terms of their ratios and numbers were minimally influenced by oral administration of CPT-11 to mice.** C57BL/6 mice of 6-8 weeks old were orally administered with CPT-11 at the dose of 400 mg/kg body weight at day 0 and day 1. (**A**) The peritoneal exudate cells were analyzed by flow cytometry at day 3. The total cell numbers were determined by a hemocytometer. (**B**) The ratios and cell numbers of large peritoneal macrophages (LPM) and small peritoneal macrophages (SPM) were calculated using a strategy as described in Figure 1, respectively. n=6; **P* < 0.05; ns, not significant.


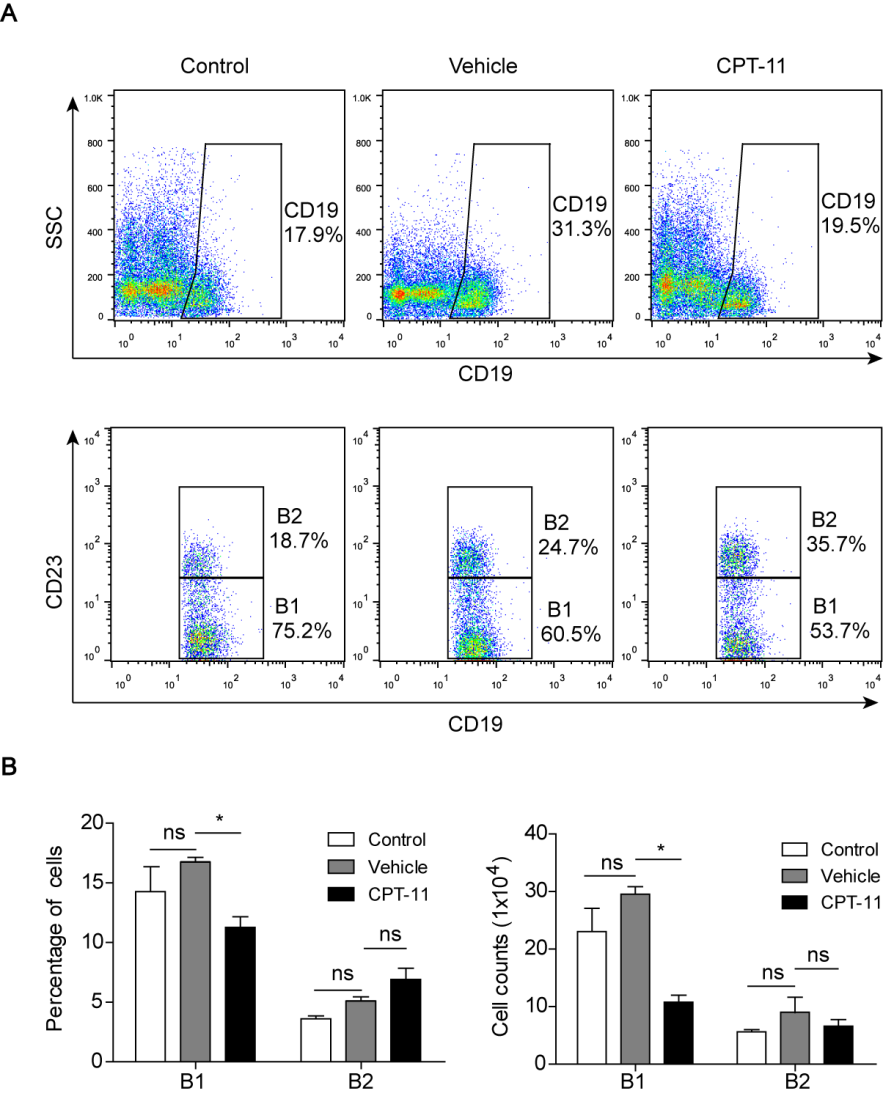


**Figure S2 ׀ Peritoneal B cells in terms of their ratios and numbers were minimally influenced by oral administration of CPT-11 to mice.** C57BL/6 mice were treated as Figure S1. The peritoneal exudate cells were analyzed by flow cytometry at day 3. The total cell numbers were determined by a hemocytometer. (**A**) A representative set of the flow cytometric data is presented. (**B**) The percentages and numbers of peritoneal B cells (including B1 and B2 cells) were calculated using a strategy as described in Figure 4. n=6; **P* < 0.05; ns, not significant.


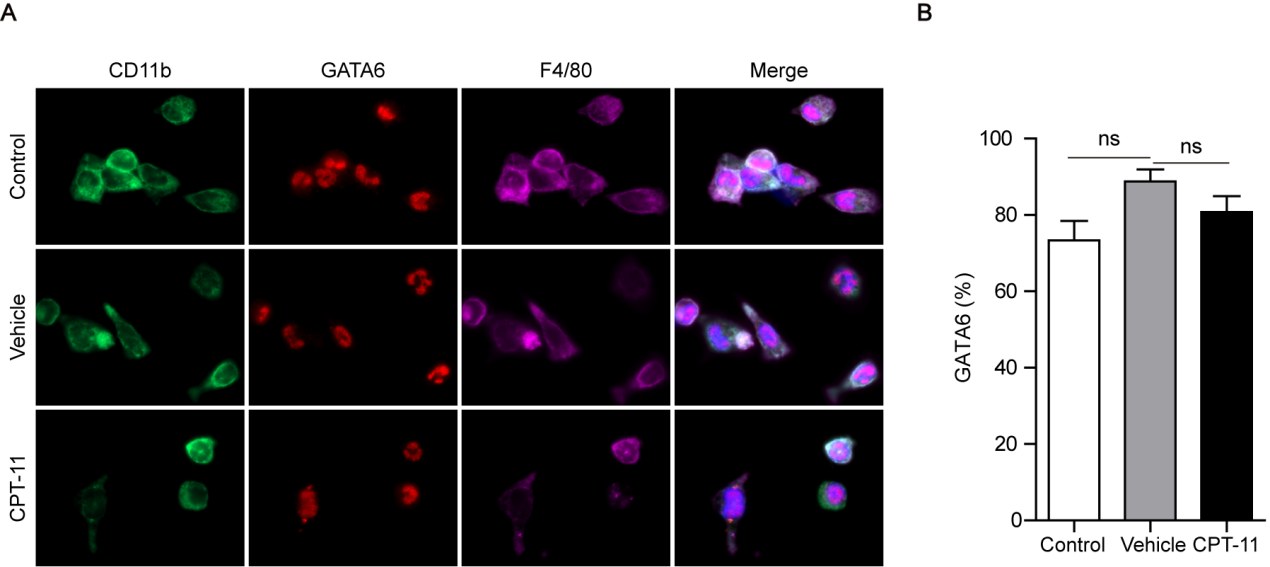


**Figure S3 ׀ Oral administration did not significantly changed the expression of GATA6 in peritoneal macrophages.** (**A**) Mice were treated as Figure S1. The peritoneal exudate cells were cultured in glass-bottomed dishes. After being stained with indicated antibodies as shown in Figure 3 and Figure 6, the cells were observed by immunofluorescence microscopy. (**B**) The percentages of peritoneal macrophages expressing GATA6 were calculated by their numbers to that of total adherent macrophages (indicating by nuclear staining with Hoechst 33342) in 10 microscopic fields. ns, not significant.


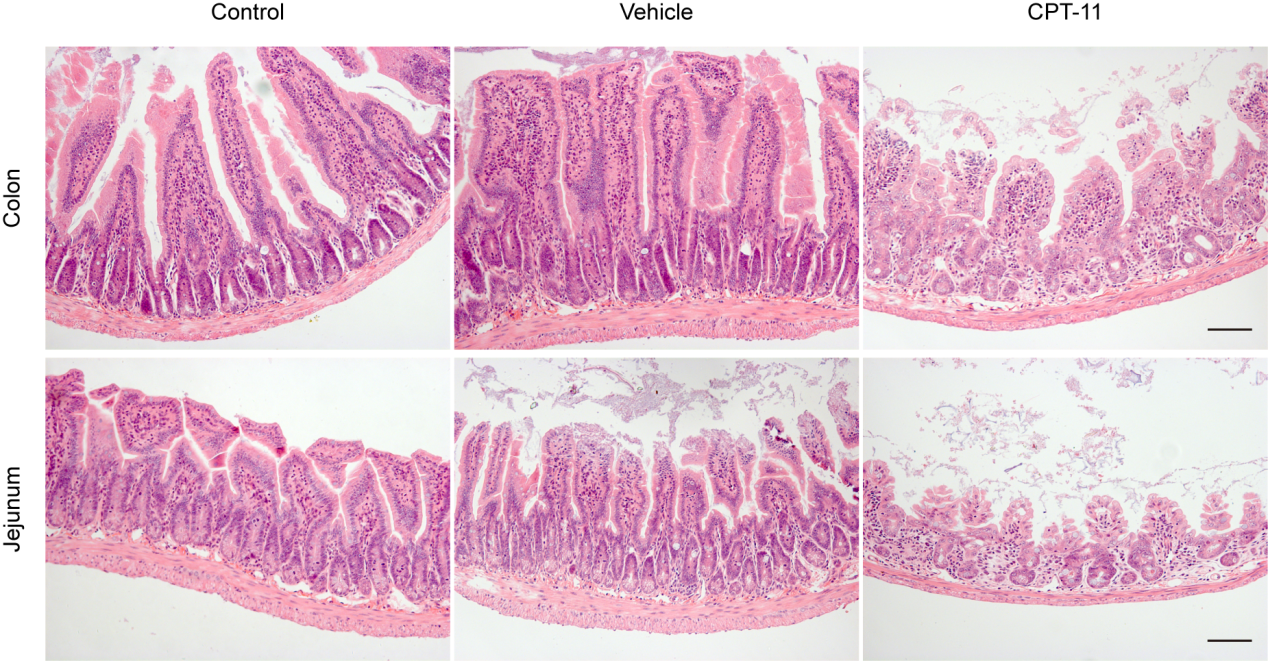


**Figure S4 ׀ CPT-11 treatment by oral route induced intestinal inflammation.** Mice were treated as indicated in Figure S1. Three days after CPT-11 treatment, the intestines were fixed in 4% neutral formaldehyde solution. The sections in paraffin wax were stained by hematoxylin-eosin. It should be noted that after CPT-11 treatment, the intestinal crypts were disappeared. But the epithelial vacuoles that were observed by naked eyes seemed having been shrunk after formaldehyde fixation in the sections of CPT-11 groups. Scale bars, 100 μm.


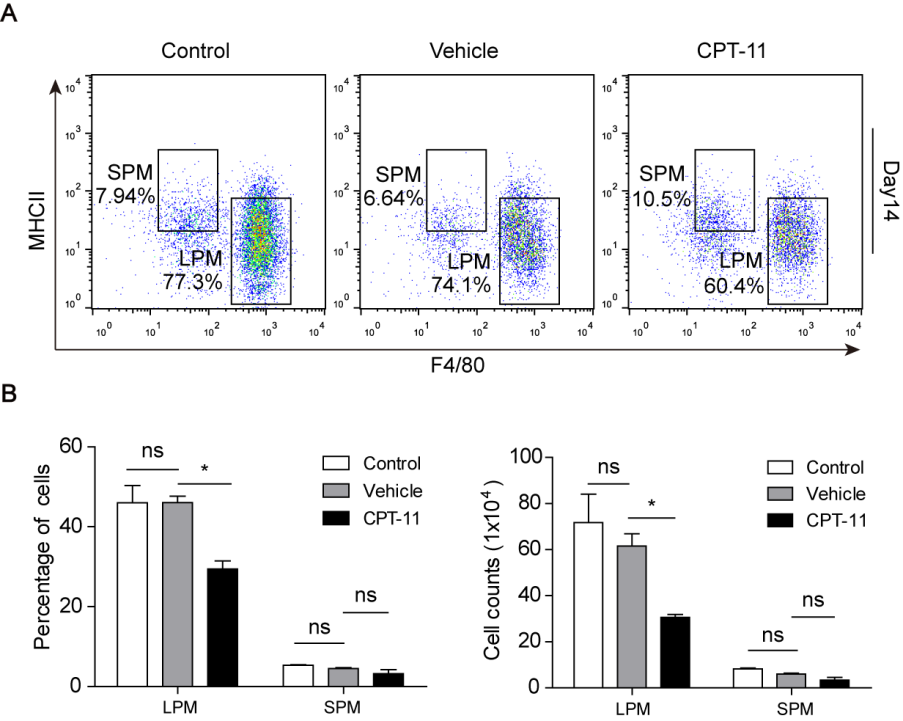


**Figure S5 ׀ The ratios of large peritoneal macrophages (LPMs) but not B cells were slightly decreased by oral CPT-11 treatment for 14 days.** C57BL/6 mice of 6-8 weeks old were orally administered with CPT-11 once at the dose of 400 mg/kg body weight at day 0. After 14 days, the mice were sacrificed and the total cell numbers in the peritoneal exudate were determined by a hemocytometer. (**A**) The ratios and cell numbers of macrophages and B cells were analyzed by flow cytometry, and a representative set of the flow cytometric data is presented. (**B**) The percentages and cell counts of indicated phenotypes in each group are calculated, respectively. n=6. **P* < 0.05; ns, not significant.


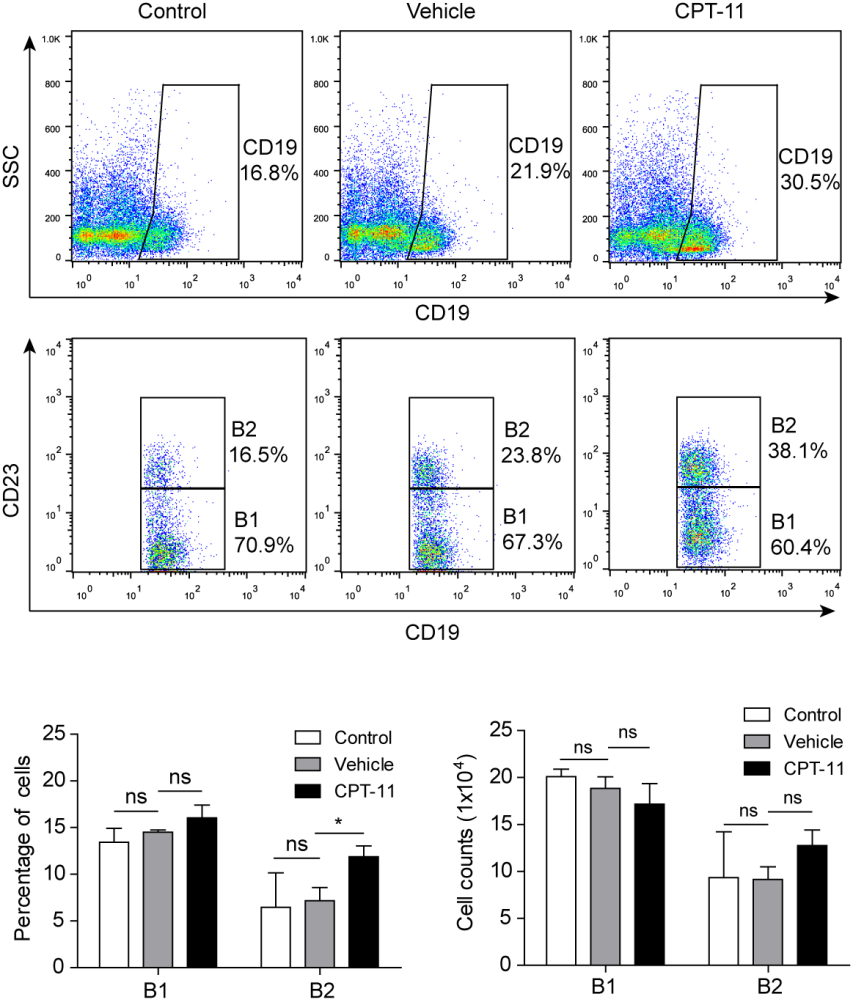


**Figure S6 ׀ Mouse peritoneal B cells in terms of their ratios and numbers were minimally influenced by oral administration of CPT-11.** C57BL/6 mice were orally treated with CPT-11 at 400 mg/kg body weight at day 0. After 14 days, the mice were sacrificed and their peritoneal exudate cells were isolated and analyzed by flow cytometry. The total cell numbers were determined by a hemocytometer. The ratios and numbers of peritoneal B cells (including B1 and B2 cells) were calculated using a strategy as described in Figure 4. A representative set of 14-days-treatment data was presented. n=6. **P* < 0.05; ns, not significant.


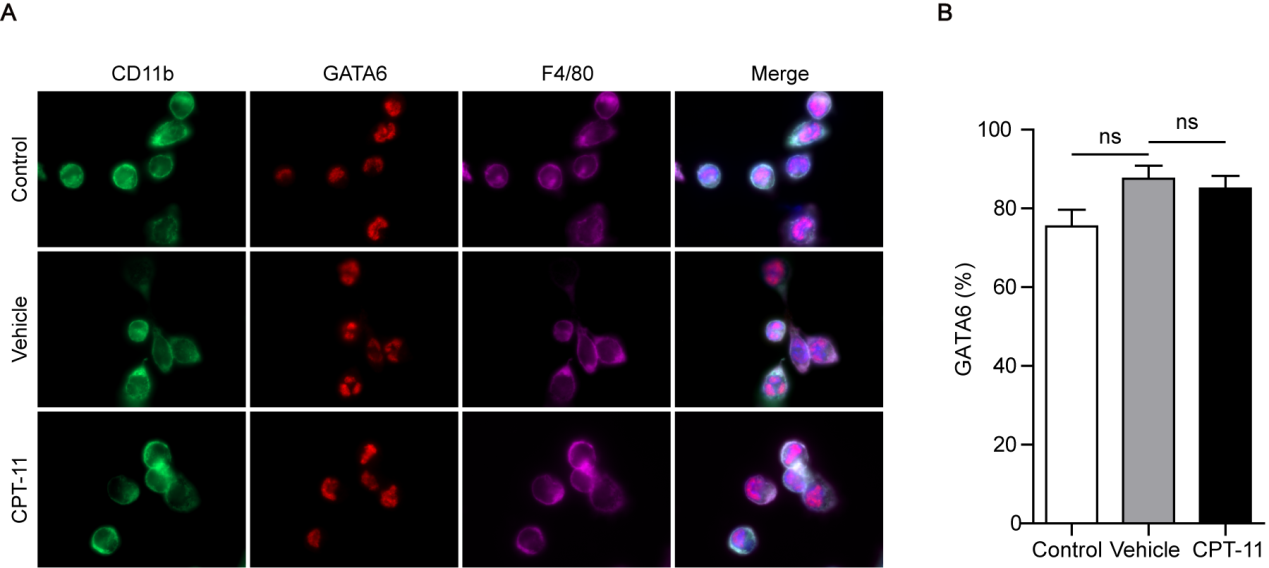


**Figure S7 ׀ Oral CPT-11 treatment did not significantly change the expression of GATA6 in peritoneal macrophages at day 14.** (**A**) Mice were orally treated with CPT-11 for 14 days. The peritoneal exudate cells were cultured in glass-bottomed dishes. After being stained with indicated antibodies as shown in Figure 3 and Figure 6, the cells were observed by immunofluorescence microscopy. A set of representative images of 14-days-treatment were presented. (**B**) The percentages of peritoneal macrophages expressing GATA6 were calculated by their numbers to that of total adherent macrophages (indicating by nuclear staining with Hoechst 33342) in 10 microscopic fields. ns, not significant.
